# Impact of COVID-19 on the HIV Treatment Outcomes Among Men Who Have Sex with Men in South Africa After the Implementation of a Differentiated Service Delivery Model: An Interrupted Time Series Analysis

**DOI:** 10.3390/ijerph22030452

**Published:** 2025-03-19

**Authors:** Betty Sebati, Edith Phalane, Yegnanew A. Shiferaw, Jacqueline Pienaar, Stanford Furamera, Refilwe Nancy Phaswana-Mafuya

**Affiliations:** 1 South Africa Medical Research Council/University of Johannesburg (SAMRC/UJ) Pan African Centre for Epidemics Research (PACER) Extramural Unit, Faculty of Health Sciences, Johannesburg 2092, South Africa; edithp@uj.ac.za (E.P.); refilwep@uj.ac.za (R.N.P.-M.); 2 Department of Statistics, University of Johannesburg, Johannesburg 2092, South Africa; yegnanews@uj.ac.za; 3 Aurum Institute, Johannesburg 2092, South Africa; jpienaar@auruminstitute.org (J.P.); sfuramera@auruminstitute.org (S.F.)

**Keywords:** COVID-19, key populations, men who have sex with men, HIV treatment outcomes, South Africa

## Abstract

The impacts of COVID-19 among men who have sex with men (MSM), who face limited access to HIV services due to stigma, discrimination, and violence, need to be assessed and quantified in terms of HIV treatment outcomes for future pandemic preparedness. This study aimed to evaluate the effects of the COVID-19 lockdown on the HIV treatment cascade among MSM in selected provinces of South Africa using routine programme data after the implementation of differentiated service delivery (DSD) models. An interrupted time series analysis was employed to observe the trends and patterns of HIV treatment outcomes among MSM in Gauteng, Mpumalanga, and KwaZulu-Natal from 1 January 2018 to 31 December 2022. Interrupted time series analysis was applied to quantify changes in the accessibility and utilisation of HIV treatment services using the R software version 4.4.1. The segmented regression models showed a decrease followed by an upward trend in all HIV treatment outcomes. After the implementation of the DSD model, significant increases in positive HIV tests (estimate = 0.001572; *p* < 0.001), linkage to HIV care (estimate = 0.001486; *p* < 0.001), ART initiations (estimate = 0.001003; *p* = 0.004), ART collection (estimate = 0.001748; *p* < 0.001), and taking viral load tests (estimate = 0.001109; *p* = 0.001) were observed. There was an overall increase in all HIV treatment outcomes during the COVID-19 lockdown in light of the DSD model.

## 1. Introduction

With nearly 8.45 million people living with HIV (PLHIV) and an estimated 164,000 new HIV cases in 2022, South Africa has the greatest HIV prevalence [1]. The HIV epidemic affects men who have sex with men (MSM) disproportionately [2,3]. Men who have sex with men have a 22 times higher risk of HIV acquisition than men in the general population aged 15 and 49 years [4]. In South Africa, the prevalence of HIV among MSM ranged from 13.2% to 58.4% between 2010 and 2019 [5].

The vulnerability of MSM to HIV is due to a variety of factors. These include biological vulnerabilities, sexual behaviours, and social and economic disadvantages, which might affect how a person obtains HIV prevention measures, testing, or treatment [6,7]. The sociocultural factors influencing the above among MSM in South Africa include the wide condemnation of homosexuality. This may result in internalised stigma, consequently leading to isolation due to fear of being socially excluded (i.e., lack of social support), violence, and discrimination from healthcare workers and the community at large [8,9]. In some cases, there is limited familiarity with and access to MSM-tailored HIV services and MSM-friendly facilities. Consequently, this influences sexual behaviour and leads to reduced access to and utilisation of these services [10,11].

Men who have sex with men may have a heightened risk of acquiring HIV and other sexually transmitted infections (STIs) due to behavioural or biological factors, including the number of concurrent partners, substance use, condomless and anal sex [6,12,13]. Consequently, they often experience negative HIV treatment outcomes such as delayed diagnosis and poor adherence to antiretroviral therapy (ART) [14,15,16]. Poor HIV treatment outcomes and ongoing transmission can lead to higher rates of HIV-related illnesses and mortality [15,16].

In addition to these persistent barriers, the COVID-19 outbreak interrupted the provision of healthcare services, including delays in HIV testing, the initiation of ART, retention in care, viral load monitoring, and CD4 count [17,18,19]. Similar impacts were reported in South Africa, with ART initiation and HIV testing being the most impacted HIV services, consequently leading to poor HIV treatment outcomes [20,21,22]. The intensity of the disruption varied and was as high as 75% in some areas [3], irrespective of the World Health Organization (WHO) issuing guidelines for the continuity of essential health services [23]. Furthermore, a study conducted in Rwanda between March and April 2020 reported that less than half of PLHIV attended their scheduled appointments for ART collection [24]. These disruptions led to HIV treatment loss to follow-up, immunological failure, virological failure, and increased mortality [18,19].

The early implementation of mitigation measures may have lessened the COVID-19 effect on HIV care delivery and utilisation. These may have included accelerating the usage of digital health platforms (i.e., eHealth), utilising private courier services for the delivery of medications and other items, or leveraging existing community networks for service delivery [25]. These are collectively referred to as the differentiated service delivery (DSD) model, which is a client-centred approach to improving the effectiveness and quality of HIV services by adapting the services to patients’ needs while lowering the burden on the healthcare system [26].

Men who have sex with men remain underserved and marginalised in HIV studies and responses, despite their high HIV acquisition and transmission rates, which contribute to the overall HIV burden [5,27,28]. The inability to address the unmet needs of MSM may negatively hamper efforts to reach the global goal of HIV epidemic control by 2030 [28]. Hence, this study aims to evaluate the effects of the COVID-19 lockdown on the HIV treatment cascade among MSM in selected provinces of South Africa using routine programme data after the implementation of DSD models.

## 2. Materials and Methods

### 2.1. Study Design

An interrupted time series analysis design was followed in analysing HIV routine programme data collected by a programme partner of the National Department of Health (NDoH).

### 2.2. Study Population and Area

The NDoH implementation partner conducted the programme among MSM (gay, bisexual men, and other MSM), aged 18 years and above, in three provinces across South Africa, namely KwaZulu-Natal (eThekwini and UMgungundlovu Districts), Gauteng (Tshwane and Ekurhuleni Districts), and Mpumalanga (Ehlanzeni District). Below is a brief contextual description of each area.

#### 2.2.1. KwaZulu-Natal (eThekwini and UMgungundlovu Districts)

With a land size of 94,361 square kilometres, KwaZulu-Natal is the third smallest of South Africa’s nine provinces. The province is the second most inhabited, with a population of about 12.3 million [29]. It consists of one metropolitan municipality (i.e., eThekwini) and 10 district municipalities, namely uMgungundlovu, Uthungulu, Amajuba, Ilembe, Sisonke, Ugu, uMkhanyakude, uMzinyathi, Uthukela, and Zululand, which are further divided into local municipalities. uMgungundlovu is the capital city of KwaZulu-Natal Province [30]. Additionally, KwaZulu-Natal is South Africa’s HIV epicentre, with a reported 16%, the second highest in the country, with eThekwini and UMgungundlovu being the leading municipalities, with the province’s highest HIV prevalences, accounting for 17.6% and 9.2% of the province’s HIV prevalence, respectively [31].

#### 2.2.2. Gauteng (Tshwane and Ekurhuleni Districts)

Gauteng is the smallest province, comprising 18,178 square kilometres of land, yet it is the most populated province. The population size is nearly 15.9 million [29]. The cities of Tshwane, Ekurhuleni, and Johannesburg are the province’s three metropolitan municipalities. It is economically diverse and large, with the City of Ekurhuleni contributing over a quarter of Gauteng’s gross domestic product [30]. The City of Johannesburg accounts for 36% of the population in Gauteng, which is approximately 5 million people. It is also known as the economic centre of South Africa. The country’s largest metropolitan municipality is the City of Tshwane, which lies in South Africa‘s capital city, Pretoria [30]. From the latest national survey, it has been reported to have an HIV prevalence of 11.9%, which is the third lowest in the country. However, the prevalence was disproportionately higher (20%) for the 25–49 years age group [32].

#### 2.2.3. Mpumalanga (Ehlanzeni District)

Mpumalanga is the second-smallest province, with a land area of 76,495 square kilometres. It has a population size of approximately 5.1 million people, making it the sixth most populated province in South Africa [29]. It is divided into three districts, namely Ehlanzeni, Gert Sibande, and Nkangala [30]. These are further divided into local municipalities. Ehlanzeni District is composed of four local municipalities, including Mbombela, the capital city of the province. The district constitutes over a third of the province’s land area [30]. Based on the latest national survey, Mpumalanga has the highest HIV prevalence of 17.4%, with Ehlanzeni accounting for 16% of the province’s HIV prevalence [33].

The target population included MSM (i.e., gay, bisexual, and other MSM) who accessed HIV treatment services from the KP MSM Prevention Programme between 1 January 2018 and 31 December 2022. A total of 96,991 MSM (308 before and 96,683 MSM during the COVID-19 lockdown) were reached by the programme in the five implementing districts across the three provinces combined. The MSM were aged 18 years and above, residing in one of the three selected provinces and districts mentioned above.

### 2.3. Data Source

The NDoH implementation partner is a large non-governmental organisation (NGO), supported by the Centres for Disease Control and Prevention (CDC), which provides HIV, tuberculosis (TB), and STI services to high-risk communities including MSM in South Africa. The KP MSM Prevention Programme was implemented in five districts of three Provinces in South Africa. The districts were selected based on having the highest HIV prevalence and incidence among key populations in South Africa. The rationale of the programme is to prevent HIV transmission and lower new cases by offering prescribed services (i.e., HIV, TB, and STI screening, treatment, and prevention).

#### Description of the DSD Model

The NGO implemented a DSD model through the KP MSM Prevention Programme to mitigate the impact of the COVID-19 pandemic on the HIV services among MSM. It was implemented within the first three months of the lockdown. The model was composed of door-to-door and community-centred HIV service provision inclusive of home-based HIV testing, ART initiations, and the multi-month dispensing of ART deliveries. The implementation of the model continued and improved with time as the lockdown restrictions were eased.

### 2.4. Research Measures

De-identified routine programme data (individual-level data), collected using mandatory NDoH data collection tools by the implementation partner of the NDoH through the KP MSM HIV Prevention Programme, were analysed. The collected information included age; location; HIV testing and prevention; ART services such as HIV counselling; HIV status; linkage to care; ART initiation; ART collection; ART adherence; ART retention; STI risk assessment such as STI screening, diagnosis, and treatment; and clinical data, e.g., creatinine, CD4 count, and viral load suppression. Data were captured into RedCap v15.0.14 and the Three Interlinked Electronic Register (Tier.net) v1.13.3.0 software, which is the NDoH data capturing platform. The data are stored in the NGO’s data warehouse for cleaning, analysis, and report generation.

For the current study, the following measures/indicators were analysed: positive HIV tests, linkage to HIV care, ART initiation, ART collection, and viral load tests. The operational definitions of the measures included in the study can be seen in Table 1 below.

### 2.5. Validity and Quality Assessment

The data were collected using an NDoH data collection tool with NDoH HIV indicators, which were informed by the National Strategic Plan 2023–2028 and the Global AIDS Monitoring (GAM) report, in addition to the HIV epidemiological context in South Africa [34,35]. This ensured the standardisation of the data collection methods across various settings, while reducing variability and increasing both the reliability and replicability of the data collection [36]. Alignment with the NSP and GAM gives this study relevance to ongoing public health interventions nationally and globally, enabling comparisons and policy relevance, including funding allocations and HIV programmatic interventions. This further improves the impact and quality of the study.

### 2.6. Statistical Analysis

Descriptive statistics were used to describe the characteristics of the target population and the changes in HIV treatment outcomes before and during the COVID-19 lockdown. To quantify changes in the HIV treatment outcomes (i.e., positive HIV tests, linkage to HIV care, ART initiation, ART collection, and viral load tests) before and during the COVID-19 lockdown, Poisson regression models were used. The interruption was the COVID-19 lockdown (March 2020), and there were two time periods (i.e., January 2018 to February 2020: before COVID-19; March 2020 to December 2022: during COVID-19 lockdown).

This study utilised an interrupted time series analysis to assess the long-term effects of the DSD model. Specifically, we employed segmented regression analysis, a robust statistical method for the estimation of the effects of interventions in interrupted time series studies [37,38], to assess changes in the utilisation of HIV treatment services and outcomes among MSM.

The analysis included terms for the onset of the COVID-19 lockdown and its interaction with time, allowing us to evaluate changes in both the pre-lockdown and lockdown periods. Additionally, we examined the significance of the coefficients in the model to determine whether the COVID-19 pandemic had a statistically significant effect on various outcome variables. All outcome variables were log-transformed to stabilise the variance and normalise the data distribution, ensuring the validity of the regression assumptions and enhancing the robustness of the analysis. Furthermore, log transformation can facilitate the interpretation of results in terms of relative changes. To assess the distributions of the HIV treatment outcomes between the two time periods (i.e., pre-COVID-19 and during the COVID-19 lockdown), we employed the Mann–Whitney U test, also known as the Wilcoxon rank-sum test. This non-parametric approach is well suited for the comparison of two independent groups when the data do not follow a normal distribution. A boxplot was computed to show the comparison of HIV treatment outcomes between the periods before and during the COVID-19 pandemic. The R software version 4.4.1 was utilised for the analysis.

### 2.7. Ethics Approval

Study approval was secured from the University of Johannesburg Research Ethics Committee (REC-1949-2023), including a waiver for informed consent. Additionally, approval to access and analyse the de-identified MSM data was approved by the NGO that collected the data (DSGC-00038). The data were saved on a password-protected online platform with controlled access at the University of Johannesburg.

## 3. Results

### Characteristics of the Study Population

Table 2 below presents the characteristics of the MSM included in the study. The majority of the MSM who accessed and utilised HIV services were from Gauteng Province, City of Tshwane Metropolitan Municipality, before the COVID-19 lockdown (217 MSM; 70.5%), while the majority were from Kwa Zulu Natal, eThekwini Metropolitan Municipality, during the COVID-19 lockdown (36,676 MSM; 37.9%). A total of 165 (98.8%) and 4273 (98.4%) MSM were linked to HIV care before and during the COVID-19 lockdown. Concerningly, the majority of the MSM, i.e., 167 (57.2%) and 55,822 (93.7%), were unable to collect their ART medication before and during the COVID-19 lockdown.

Table 3 presents the first quartile (Q1), median, third quartile (Q3), and interquartile range (IQR) of HIV treatment outcomes among MSM. All outcomes showed similar patterns of drastic increases in the median from before to during the COVID-19 lockdown, with ART initiation having a median range of 2 to 125.50, ART collections from 4 to 114.00, and viral load tests from 5 to 41.50. The IQR also increased from before to during the COVID-19 lockdown as follows: 1.75–86.25 for ART initiation, 4–89.00 for ART collection, and, lastly, 3.00–31.00 for viral load tests, which was the lowest IQR observed. There was an upward distribution of values before and during the COVID-19 lockdown (i.e., ART initiation: 0.25–2 and 76.75–163.00; ART collection: 2–6 and 65.75–154.75, and viral load tests: 3–6 and 22.00–53.00).

**Figure 1:**
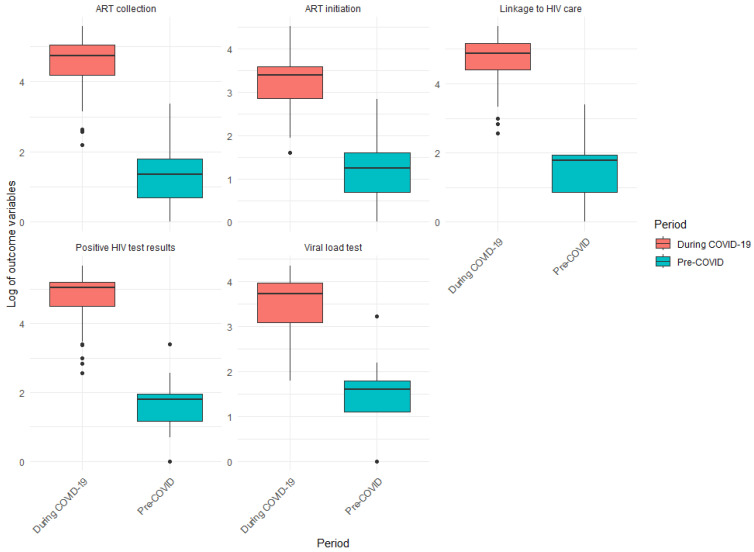
Comparison of HIV treatment outcomes, i.e., before and during the COVID-19 lockdown.

Figure 1 presents a boxplot showing the comparison of the HIV treatment outcomes from before and during the COVID-19 pandemic. The boxplot highlights an increase in ART collection, ART initiation, linkage to HIV care, positive HIV test results, and viral load testing during the COVID-19 lockdown. The median values during the COVID-19 lockdown are consistently higher, indicating that access to HIV services was more limited before the pandemic. Furthermore, the wider interquartile ranges (IQRs) suggest greater variability in service access. Overall, it seems that the COVID-19 pandemic has resulted in increased engagement in these services.

Table 4 illustrates the results of the Mann–Whitney U test, which was used to evaluate the distributions of each HIV treatment outcome before and during the COVID-19 lockdown. The results demonstrate that all outcome variables—positive HIV tests, linkage to HIV care, ART initiation, ART collection, and viral load tests—experienced significant increases during the COVID-19 lockdown compared to before (*p* < 0.001 for all outcome variables). This indicates that the COVID-19 lockdown coincided with heightened activity and engagement in HIV-related services. This aligns with the results shown in Table 3, which indicate that the median values during the COVID-19 lockdown were significantly higher than before the COVID-19 lockdown for all outcome variables.

**Figure 2:**
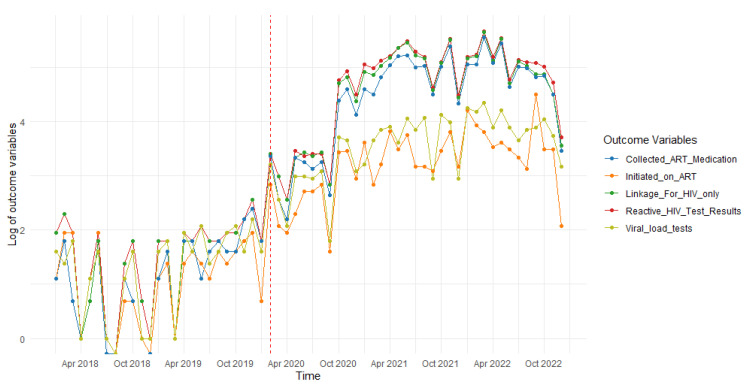
The time series plots illustrate the trends in positive HIV tests, linkage to HIV care, ART initiation, ART collection, and viral load testing among MSM before and during the COVID-19 pandemic. The month when interruptions occurred is marked as March 2020, indicated by a dotted vertical line on the graph. Table 5 below further quantifies these trends.

Figure 2 shows the time series plots of the HIV treatment outcomes, including positive HIV tests, linkage to HIV care, ART initiation, ART collection, and viral load tests. All these outcomes exhibit similar trends, revealing a downward trajectory in the years leading up to the COVID-19 lockdown (January 2018 to February 2020).

With the onset of the COVID-19 lockdown in March 2020 (indicated by the vertical dotted line on the graphs), there was an initial decline followed by an upward trend across all outcomes. This pattern resulted in alternating periods of decline and growth. Nevertheless, there was an overall increase in all outcomes during the COVID-19 lockdown compared to the period before.

Table 5 below shows the interrupted time series models predicting changes in HIV treatment outcomes from January 2018 to December 2022 (i.e., positive HIV tests, linkage to HIV care, ART initiation, ART collection, and viral load tests).

Positive HIV tests

The introduction of the COVID-19 lockdown had a significant negative impact, resulting in a decrease of 26.79 (95% CI: −42.17, −11.40; *p* = 0.010) in the logarithm of “positive HIV tests”. Finally, after the onset of the COVID-19 lockdown, the logarithm of “positive HIV tests” increased by 0.001572 (95% CI: 0.00075, 0.00239; *p* < 0.001). Additionally, the adjusted R-squared value is 0.9648, indicating that the model explains 96.48% of the variability in the log-transformed outcome variable.

Linkage to HIV care

The onset of the COVID-19 lockdown had a significant negative impact, leading to a substantial decrease of 25.21 (95%CI: −40.70, −9.71; *p* = 0.002) in the logarithm of “linkage to HIV care”. After the onset of the COVID-19 lockdown, the logarithm of “linkage to care” increased by 0.001486 (95%CI: 0.00066, 0.00231; *p* < 0.001). The adjusted R-squared value is 0.9634, suggesting that the model explains 96.34% of the variability in the log-transformed outcome variable.

ART initiation

The COVID-19 lockdown significantly impacted ART initiations, resulting in an estimated proportional decrease of 17.10 (95% CI: −29.92, −4.29; *p* = 0.009). Conversely, the time elapsed since the lockdown began shows a significant effect, leading to a proportional increase of 0.1003% (95% CI: 0.00032, 0.00168; *p* = 0.004) in ART initiations for each additional time unit. The adjusted R-squared value is 0.952, indicating that the model explains 95.2% of the variability in the log-transformed outcome variable.

ART collection

The COVID-19 lockdown significantly impacted ART collection, resulting in an estimated proportional decrease of 30.00 (95% CI: −45.87, −14.13; *p* < 0.001). Conversely, the time elapsed since the lockdown began shows a significant effect, leading to a proportional increase of 0.1748% (95% CI: 0.00091, 0.00259; *p* < 0.001) in ART collection for each additional time unit. The adjusted R-squared value is 0.9582, indicating that the model explains 95.82% of the variability in the log-transformed outcome variable.

Viral load tests

The COVID-19 lockdown significantly impacted the viral load tests performed, resulting in an estimated proportional decrease of 19.00 (95% CI: −31.39, −6.61; *p* = 0.003). Conversely, the time elapsed since the lockdown began shows a significant effect, leading to a proportional increase of 0.1109% (95% CI: 0.00045, 0.00177; *p* = 0.001) in the viral load tests performed for each additional time unit. The adjusted R-squared value is 0.9626, indicating that the model explains 96.26% of the variability in the log-transformed outcome variable.

## 4. Discussion

The aim of this study was to evaluate the impact of the COVID-19 lockdown on the HIV treatment cascade among MSM in selected provinces of South Africa using routine programme data after the implementation of the DSD model. The majority of the MSM accessing the KP MSM Prevention Programme during the COVID-19 lockdown were from Kwa Zulu Natal, eThekwini Metropolitan Municipality (36676 MSM; 37,9%). During the COVID-19 pandemic, an overall increase in trend was observed for all HIV treatment indicators, i.e., positive HIV tests, linkage to HIV care, ART initiations and collections, and viral load testing. Similar results were shown by the Mann–Whitney U test across all outcomes.

In South Africa, some of the measures implemented to manage the virus’s spread included movement restrictions, maintaining physical distancing, and the closure of public places [39]. Only those that offered essential services, such as healthcare, were allowed to operate [40]. Similar measures were implemented in other countries and were reported to have further isolated and disproportionately affected KPs, exposing them to more discrimination and stigma [41]. Although the negative effects of the COVID-19 lockdown on the HIV treatment outcomes among MSM in the current study did not persist, there is a possibility of negative impacts on their social and sexual life [42]. This was noted in a study by Cascalheira et al. [42], who reported a disconnect in the latter among MSM and worsening health consequences for PLHIV in the US. A South African study reported increased risky sexual behaviours among youth, particularly men, during the pandemic [43].

A South African study by Yao et al. [44], conducted among KPs (i.e., FSW, MSM, and transgender women), reported 13,593 and 2771 confirmed new HIV diagnoses before and during the high-restriction COVID-19 lockdown, respectively. Additionally, 10,687 new HIV diagnoses were observed in the less restricted period of the COVID-19 lockdown. Contrastingly, this study observed more positive HIV tests during the lockdown than before. This could be a result of reduced access to HIV preventative services during the initial stages of the COVID-19 lockdown, leading to increased HIV transmission rates [45].

It may also be a result of interventions implemented to increase access to HIV services during this period, similarly to another study [21], wherein more MSM obtained access to HIV tests. Although the programme in this study provided HIV tests prior to the pandemic, innovative measures to reach more MSM were implemented and intensified as a result of the pandemic. The sustained implementation of innovative service delivery such as DSD models lessened the pandemic’s impact on health service provision and accessibility through longer ART dispensing intervals, enabling PLHIV to wait longer without visiting health facilities, among other measures [46,47].

Evidence from the WHO has shown that DSD models improve both the quality of care and health outcomes of PLHIV, while ensuring the effective functioning of health systems. Furthermore, this model allows the health system to redirect resources towards the most vulnerable [48]. The DSD model empowers those targeted (i.e., MSM) to find a mode of HIV care that is favourable to their lifestyle. Limited resources are maximised by tailoring health services to the individual’s preferences and clinical requirements, as well as to the local context [49]. The current study implemented DSD models to ensure the latter and mitigate the impact of the COVID-19 lockdown. These included door-to-door and community-centred HIV service provision to the MSM community (i.e., home-based ART initiations and deliveries including multi-month dispensing). However, despite the above, a noticeable decline in all the outcomes, mostly ART initiations and viral load tests, was observed from October 2022, as depicted in Figure 2. This could be due to the ending of the financial year for the KP MSM Prevention Programme, resulting in disruptions to the delivery of these services, mostly affecting the reach of new clients.

As with Siedner et al. [50] and Dorward et al. [20], HIV service outcomes, including the number of HIV tests performed, positive HIV results, and ART adherence, immediately decreased at the start of the COVID-19 lockdown, as depicted by the segmented regression models. This was followed by an increase in these services/outcomes and some variability wherein all outcomes generally improved. This could have been due to the easing of the lockdown restrictions and service providers putting measures in place to ensure service continuity [21]. Another study reported the use of virtual support platforms on social media and short message services (SMS) to continuously engage with MSM on the availability of and access to HIV services, with improvements in some of the services, including an increased number of HIV tests conducted and positive HIV results [44]. Contrastingly, a significant decline in HIV services in the public sector, particularly HIV testing, was observed in a study conducted among the general population across all nine provinces of South Africa [25]. This could illustrate the disparities in access to HIV care [21] and the redirection of resources towards fighting the COVID-19 pandemic.

### 4.1. Practical Implications

Over time, the HIV treatment outcomes (i.e., positive HIV tests, linkage to HIV care, ART initiation and collection, and viral load testing) increased. Initially, the COVID-19 lockdown may have suppressed these HIV treatment outcomes. However, signs of recovery became evident over time as the DSD model was implemented. Policymakers and healthcare professionals need to prepare for these initial setbacks and put mitigating measures in place, including resource allocation, focused outreach, or additional assistance throughout the transition. The results emphasise the significance of ongoing observation/monitoring using techniques such as interrupted time series analysis. It further provides an opportunity for the necessary adjustments to be made. In this regard, stakeholders can identify early indicators of recovery and make the required adjustments to speed up favourable outcomes. Hence, the long-term benefits must be considered in addition to the short-term outcomes.

### 4.2. Strengths and Limitations of the Study

The methods employed in collecting routine programme data allow for the tracking of changes in indicators/study measures over time (i.e., time series analysis) and provide opportunities for comparisons of the methods used over time. The results could be impacted by unmeasured variables such as changes in health-seeking behaviour. As a result, the overestimation of the COVID-19 impact is possible if unmeasured factors worsened the HIV treatment outcomes during the pandemic. The data quality may have been negatively impacted due to COVID-19 disruptions, leading to missing or inaccurate data. The data were collected from multiple districts and provinces in South Africa; hence, they can provide insights for the rest of the country since it experienced similar COVID-19 conditions. Overall, the findings of this study make a vital contribution to understanding the impact of COVID-19 on HIV services. The current study can serve as a guiding tool for HIV programme service providers and policymakers in both the private and public sectors. It can assist in making informed plans to end HIV as a pandemic and ensure service continuity amid movement-restricted conditions such as COVID-19.

## 5. Conclusions

There was an overall improvement in HIV treatment outcomes among MSM during the COVID-19 lockdown. The implementation of DSD models during the restricted COVID-19 lockdown and the easing of the lockdown ensured the continuity of HIV services in the KP MSM Prevention Programme. This study adds a valuable contribution to the body of knowledge on the context-specific impact of the COVID-19 lockdown and its restrictive conditions on the access to HIV treatment services among MSM in selected districts across the three provinces of South Africa. Future studies should investigate the consistent implementation of DSD models by the private health sector in districts characterised by high HIV prevalence and incidence among key populations. This should be widely explored to assist in reaching the 95-95-95 UNAIDS goals and beyond. There is also a need to determine the impact of COVID-19 on health-seeking behaviours among key populations as this may affect HIV treatment outcomes.

## Figures and Tables

**Table 1:** Operational definitions of the measures included in the current study.

Measure	Operational Definition
Consent for HIV testing	The process of pre-counselling, informing the MSM about HIV tests, including the potential risks, benefits, and outcomes and letting them decide if they would like to continue taking the test or not.
Positive HIV tests	HIV tests that were reactive and underwent confirmatory tests.
Linkage to HIV care	The process of connecting MSM with confirmed reactive HIV tests for healthcare to begin taking treatment for their HIV infection.
ART initiation	The process of starting to receive HIV treatment after being diagnosed with HIV.
ART collection	The ability of HIV-diagnosed MSM to collect their HIV medication from the healthcare provider/facility/relevant party as required.
Viral load test	Whether MSM living with HIV were able to undertake a viral load test, regardless of their viral load/viral suppression state.
Age	Age at last birthday taken on the day of data collection.
Location	The geographical district where the MSM included in the study resided.

**Table 2:** Characteristics of men who have sex with men included in the study before and during the COVID-19 lockdown.

	Before COVID-19 Lockdown (January 2018–February 2020)	During COVID-19 Lockdown (March 2020–December 2022)	January 2018–December 2022
	n (%)	n (%)	Total
Median age (years)	31 (25–38)	28 (23–34)	30 (23–38)
District GP City of Tshwane Metropolitan Municipality GP Ekurhuleni Metropolitan Municipality KZ eThekwini Metropolitan Municipality KZ uMgungundlovu District Municipality MP Ehlanzeni District Municipality	217 (70.5) 40 (13.0) 24 (7.8) 11 (3.6) 16 (5.2)	11,930 (12.3) 32,729 (33.9) 36,676 (37.9) 6159 (6.4) 9187 (9.5)	12,147 (12.5) 32,769 (33.8) 36,700 (37.8) 6170 (6.4) 9203 (9.5) 96,989 (100)
Consent for HIV testing No Yes	3 (1.0) 305 (99.0)	28,030 (29.0) 68,652 (71.0)	28,033 (28.9) 68,957 (71.1) 96,990 (100)
HIV test result Negative Positive	125 (42.8) 167 (57.2)	40,394 (89.8) 4572 (10.2)	40,519 (89.5) 4739 (10.5) 45,258 (100)
Linkage to HIV care Yes No	165 (98.8) 2 (0.6)	4273 (98.4) 70 (1.6)	4438 (98.4) 72 (1.6) 4510 (100)
ART initiation No Yes	221 (75.7) 71 (24.3)	55,704 (93.3) 4014 (6.7)	55,925 (93.2) 4085 (6.8) 60,010 (100)
ART collection No Yes	167 (57.2) 125 (42.8)	55,822 (93.7) 3748 (6.3)	55,989 (93.5) 3873 (6.5) 59,862 (100)
Viral load test done 12 months 6 months Other	127 (97.0) 2 (1.5) 2 (1.5)	919 (67.7) 293 (21.6) 145 (10.7)	1046 (70.3) 295 (19.8) 147 (9.9) 1488 (100)

ART: antiretroviral therapy; GP: Gauteng Province; KZ: Kwa Zulu Natal; MP: Mpumalanga.

**Table 3:** Median and interquartile range of HIV treatment outcomes before and during the COVID-19 lockdown among men who have sex with men in selected provinces of South Africa.

	Before COVID-19 Lockdown				During COVID-19 Lockdown			
Outcome Variable	Q1	Median	Q3	IQR	Q1	Median	Q3	IQR
Positive HIV test results	3.25	6.00	7.00	3.75	89.25	154.00	181.00	91.75
Linkage to HIV care	2.50	6.00	7.00	4.50	81.25	132.00	176.50	95.25
ART initiation	0.25	2.00	2.00	1.75	76.75	125.50	163.00	86.25
ART collection	2.00	4.00	6.00	4.00	65.75	114.00	154.75	89.00
Viral load test	3.00	5.00	6.00	3.00	22.00	41.50	53.00	31.00

ART: antiretroviral therapy; Q1: first quartile; Q3: third quartile; IQR: interquartile range.

**Table 4:** Comparison of HIV treatment outcomes before and during the COVID-19 period using the Mann–Whitney U test.

Outcome Variable	W	*p*-Value
Positive HIV test results	5.50	<0.001
Linkage to HIV care	5.50	<0.001
ART initiation	16.5	<0.001
ART collection	8.50	<0.001
Viral load test	19.5	<0.001

**Table 5:** Interrupted time series models predicting changes in HIV treatment outcomes from January 2018 to December 2022 among men who have sex with men in South Africa.

HIV Treatment Outcome	Time Before the COVID-19 Lockdown	COVID-19 Lockdown	Time After the COVID-19 Lockdown
Positive HIV tests			
Estimate	0.00009885 (0.0000833, 0.000114)	−26.79 (−42.17, −11.40)	0.001572 (0.00075, 0.00239)
Standard error	0.000007777	7.69	0.0004082
*t*-value	12.71	−3.49	3.85
*p*-value	<0.001	0.010	<0.001
Adjusted R-squared	0.9648		
Linkage to HIV care			
Estimate	0.00009767 (0.0000820, 0.000113)	−25.21 (−40.70, −9.71)	0.001486 (0.00066, 0.00231)
Standard error	0.000007842	7.737	0.000411
*t*-value	12.47	−3.26	3.62
*p*-value	<0.001	0.002	<0.001
Adjusted R-squared	0.9634		
ART initiation			
Estimate	0.00007869 (0.0000657, 0.0000917)	−17.10 (−29.92, −4.29)	0.001003 (0.00032, 0.00168)
Standard error	0.000006478	6.4	0.00034
*t*-value	12.15	−2.67	2.95
*p*-value	<0.001	0.009	0.004
Adjusted R-squared	0.952		
ART collection			
Estimate	0.00008209 (0.000066, 0.0000982)	−30.00 (−45.87, −14.13)	0.001748 (0.00091, 0.00259)
Standard error	0.000008022	7.925	0.000421
*t*-value	10.23	−3.79	4.15
*p*-value	<0.001	<0.001	<0.001
Adjusted R-squared	0.9582		
Viral load tests			
Estimate	0.00008998 (0.0000774, 0.000103)	−19.00 (−31.39, −6.61)	0.001109 (0.00045, 0.00177)
Standard error	0.000006262	6.186	0.0003286
*t*-value	14.37	−3.07	3.38
*p*-value	<0.001	0.003	0.001
Adjusted R-squared	0.9626		

ART: antiretroviral therapy.

## Data Availability

Data were obtained from a third party (NGO) and are available from the authors with the permission of the NGO.

## Declaration

An abstract derived from this manuscript was presented at the 6th National Big Data Health Science Conference held in South Carolina, Columbia, on 13th–14th February 2025. The abstract will be published as a conference proceeding in an MDPI journal, entitled as follows: “Betty Sebati, Edith Phalane, Jacqueline Pienaar, Stanford Furamera, Yegnanew Shiferaw, and Refilwe Nancy Phaswana-Mafuya. Impacts of COVID-19 Pandemic on HIV Treatment Outcomes Among Men Who Have Sex with Men in South Africa Using Routine Programme Data: An Interrupted Time Series Analysis. Poster session presented at: The 6th National Big Data Health Science Conference; 13–14 February 2025, South Carolina, Columbia”. The current manuscript includes in-depth information, i.e., data analysis, findings, and discussions, that was not included in the conference abstract.

## Abbreviations

The following abbreviations are used in this manuscript:

MSM	Men who have sex with men
COVID-19	Coronavirus disease 2019
DSD	Differentiated Service Delivery Model
ART	Antiretroviral Therapy
PLHIV	People living with HIV
KPs	Key populations
FSW	Female sex workers
PWUD	People who use drugs
PWID	People who inject drugs
TG	Transgender
SSA	Sub-Saharan Africa
CD4	Cluster of differentiation 4
STI	Sexually transmitted infection
NGO	Non-government organization
CDC	Centres for Disease Control and Prevention
NDoH	National Department of Health
SMS	Short message service
